# The interactions between traditional Chinese medicine and gut microbiota: Global research status and trends

**DOI:** 10.3389/fcimb.2022.1005730

**Published:** 2022-09-12

**Authors:** Shanshan Yang, Shaodong Hao, Qin Wang, Yanni Lou, Liqun Jia, Dongmei Chen

**Affiliations:** ^1^ Oncology Department of Integrated Traditional Chinese and Western Medicine, China-Japan Friendship Hospital, Beijing, China; ^2^ Graduate School, Beijing University of Chinese Medicine, Beijing, China; ^3^ Sixth Clinical School of Medicine, Beijing University of Chinese Medicine, Beijing, China

**Keywords:** traditional Chinese medicine, gut microbiota, correlation, research status and trends, highly cited papers, bibliometrics

## Abstract

**Background:**

There is a crosstalk between traditional Chinese medicine (TCM) and gut microbiota (GM), many articles have studied and discussed the relationship between the two. The purpose of this study is to use bibliometric analysis to explore the research status and development trends of the TCM/GM research, identify and analyze the highly cited papers relating to the TCM/GM.

**Methods:**

A literature search regarding TCM/GM publications from 2004 to 2021 was undertaken on August 13, 2022. The main information (full record and cited references) of publications was extracted from the Science Citation Index Expanded (SCI-E) of Web of Science Core Collection (WoSCC). The Bibliometrix of R package, CiteSpace and VOSviewer were used for bibliometric analysis.

**Results:**

A total of 830 papers were included. The publication years of papers were from 2004 to 2021. The number of papers had increased rapidly since 2018. China had the most publications and made most contributions to this field. Nanjing University of Chinese Medicine and Beijing University of Chinese Medicine were in the leading productive position in TCM/GM research, Chinese Academy of Chinese Medical Sciences had the highest total citations (TC). Duan Jin-ao from Nanjing University of Chinese Medicine had the largest number of publications, and Tong Xiao-lin from China Academy of Chinese Medical Sciences had the most TC. The *Journal of Ethnopharmacology* had the most published papers and the most TC. The main themes in TCM/GM included the role of GM in TCM treatment of glucolipid metabolism diseases and lower gastrointestinal diseases; the mechanism of interactions between GM and TCM to treat diseases; the links between TCM/GM and metabolism; and the relationship between GM and oral bioavailability of TCM.

**Conclusion:**

This study gained insight into the research status, hotspots and trends of global TCM/GM research, identified the most cited articles in TCM/GM and analyzed their characteristics, which may inform clinical researchers and practitioners’ future directions.

## 1 Introduction

Traditional Chinese medicine (TCM), as one of the treatment methods in East Asia for thousands of years, has attracted more and more attention due to its good efficacy and fewer side effects (Feng et al., 2021; Lin et al., 2021; Yang et al., 2022). However, due to the controversial theory of TCM, the complexity in the mechanism of TCM, and the unclearness of effective bioactive components, there are still some doubts and misunderstandings about TCM, resulting in the stagnation of research focusing on the development of TCM (Zheng et al., 2020; Che et al., 2022). Gut microbiota (GM) is involved in various metabolic processes in the human body and plays a major role in host immune response (Wang et al., 2018). GM and its metabolites are of great significance to maintain host health and the pathogenesis, prevention, and treatment of diseases (Clemente et al., 2012; David et al., 2014; Canfora et al., 2015). In recent years, GM has become an important frontier and hot topic to understand the development and progress of diseases.

With this trend, more and more TCM research began to pay attention to GM and provide rich information for TCM researchers (Li et al., 2009; Xu et al., 2017). GM has become a new way of understanding TCM and can elucidate the profound theory of TCM, which is regarded as the golden key to unlock the mystery of TCM (Chen et al., 2016; Feng et al., 2019; Yue et al., 2019; Lin et al., 2021). The crosstalk between GM and TCM is a crucial discovery in life science and a large number of studies have emerged in the last decade (Xu et al., 2017; Zhang et al., 2020; Li et al., 2021). The interactions between GM and TCM mainly include two aspects (An et al., 2019; Dey, 2019). On the one hand, TCM including single compounds, single herbs and herbal formulations can modulate the GM and its metabolites, and reverse the abnormal GM composition (Zhang et al., 2020). On the other hand, GM also has a very important impact on the biotransformation, bioactivity and bioavailability of TCM, thereby affecting its efficacy and toxicity (Yan et al., 2018; Dey, 2019; Feng et al., 2021).

Bibliometric analysis is a method of statistically evaluating the research status, development trends, and the most influential studies in a specific field. Citation analysis is one of the main methods of bibliometrics, which can evaluate the quality and recognition of papers, and better understand the discipline construction and development of a field. At present, many TCM-related areas have been well studied and explored through bibliometric analysis, such as the analysis of research trends on Artemisinin (Dong et al., 2022), TCM nursing technology for insomnia (Wang et al., 2022), acupuncture research about migraine (He et al., 2022), and traditional Chinese health exercises for improving cognitive function (Li et al., 2021). However, there is currently no English literature published on the quantitative analysis of interactions between GM and TCM. After reviewing the retrieving literature, we found that relevant studies appeared and gradually increased since 2004. Therefore, we selected the published papers from 2004 to 2021 for analysis. This article aims to identify the related papers in TCM/GM in recent years and analyze their characteristics, review the regulatory role of the GM in TCM, looking forward to providing references for further research in TCM/GM.

## 2 Materials and methods

### 2.1 Data source and search strategy

Web of Science Core Collection (WoSCC) is an important database for obtaining global academic information with a strict screening mechanism and only includes important academic journals in various disciplines in bibliometrics. SCI-E of WoSCC includes the most authoritative and influential mainstream academic journals in natural science, which is considered the best database and has been used extensively in previous bibliometric studies (Li et al., 2021; Wu et al., 2021; Cheng et al., 2022). Therefore, we choose it as the search source.

All searches were performed on the same day (August 13, 2022) to avoid the significant bias caused by database updates. The papers were retrieved from the SCI-E of WoSCC on August 13, 2022. Using the subject term “advanced search” method, the search terms were TS= “Gut Microflora” and “traditional Chinese medicine” and their synonyms (**Table 1**). Terms related to GM or TCM that entered into the WoS engine were extracted from the Medical Subject Headings (MeSH) from PubMed. The selection criteria were as follows: (1) The publication years were from 2004 to 2021; (2) The document types were limited to “article” and “review”; (3) The language type is set to English. After screening, a total of 830 papers were obtained (**Table 1**), of which 688 were “articles” and 142 were “reviews”. Two researchers (SY and SH) independently performed the search and data extraction. We extracted the information such as titles, authors, institutions, countries, publication years, keywords and so on, and saved it in text format.

**Table 1:** Search quires and refinement procedure.

Set	Results	Refinement
1	1087	Query formulation: Step 1: #1 TI OR AB OR AK =(“Gut Microbi*” OR “Gut Microflora” OR “Gut Flora” OR “Gut Microbial Flora” OR “Gut Microecology” OR “Intestinal Microbi*” OR “Intestinal Microflora” OR “Intestinal Flora” OR “Intestinal Microbial Flora” OR “Intestinal Microecology” OR “Gastrointestinal Microbi*” OR “Gastrointestinal Microflora” OR “Gastrointestinal Flora” OR “Gastrointestinal Microbial Flora” OR “Gastrointestinal Microbial Communit*” OR “Gastrointestinal Microecology” OR “Fecal Microbi*” OR “Fecal Microflora” OR “Fecal Flora” OR “Fecal Microbial Flora” OR “Faecal Microbi*” OR “Faecal Microflora” OR “Faecal Flora” OR “Faecal Microbial Flora” OR “Gut Bacteri*” OR “Intestinal Bacteri*” OR “Gastrointestinal Bacteri*” OR “Fecal Bacteri*” OR “Faecal Bacteri*” OR “Enteric Bacteri*”) Step 2: #2 TI OR AB OR AK =(“Chinese medicine*” OR “Chinese herb*” or “Chinese drug*” OR “Chinese herbal medicine*” OR “Chinese medicinal herb*” OR “Chinese herbal drug*” OR “traditional Chinese medicine*” OR “TCM*” OR “Chinese medicinal plant*” OR “Chinese materia medica” OR “Chinese decoction” OR “Chinese formula” OR “Chinese prescription*” OR “Chinese patent medicine*” OR “Chinese herbal formula*” OR “Chinese herbal compound*” OR “Chinese herbal prescription*” OR “Chinese herbal ingredient*” OR “Chinese Herbal Preparation*” OR “Herbal medicine*” OR “Traditional medicine*”) Step 3: #1 AND #2 Indexes =SCI-EXPANDED
2	858	Refined by PUBLICATION YEARS: (2004-2021)
3	854	Refined by LANGUAGES: (ENGLISH)
4	830	Refined by DOCUMENT TYPES: (ARTICLES OR REVIEW ARTICLES)

The wildcard "*" was used in place of any number of characters for the most comprehensive search of relevant literature.

### 2.2 Data analysis and parameter query

Bibliometrix analysis was performed using a specific program from Bibliometrix R package based on Rstudio (version 2022.03.10, RStudio team, Boston, MA, USA), CiteSpace (version 6.1.R2), VOSviewer (version 1.6.18, Leiden University Science and Technology Research Center, The Netherlands) and Microsoft Excel 2019 (Microsoft, Redmond, Washington, USA). VOSviewer and CiteSpace are developed for building and visualizing bibliometric networks. The Bibliometrix R package provides a suite of tools for quantitative research in scientometrics. Each software allows for the construction and visualization of bibliometric networks to facilitate understanding of TCM/GM. Specifically, the distribution of each component analyzed in the bibliometric analysis was assessed by a software package applying machine learning. For this, we used the following variables: annual scientific production, average citations per year, most relevant sources, source dynamics, most local impact source by H-index or total citations (TC), most relevant authors, top authors’ production over time, author local impact, country scientific production, most relevant affiliations, collaboration network by countries, corresponding author’s country, historical direct citation network, most global cited documents, most relevant keywords, cluster analysis. The journal impact factor (IF) and partition can be found in the “Journal Citation Reports ™ 2021”.

## 3 Results

### 3.1 Annual publication trends in TCM/GM

**Figure 1:**
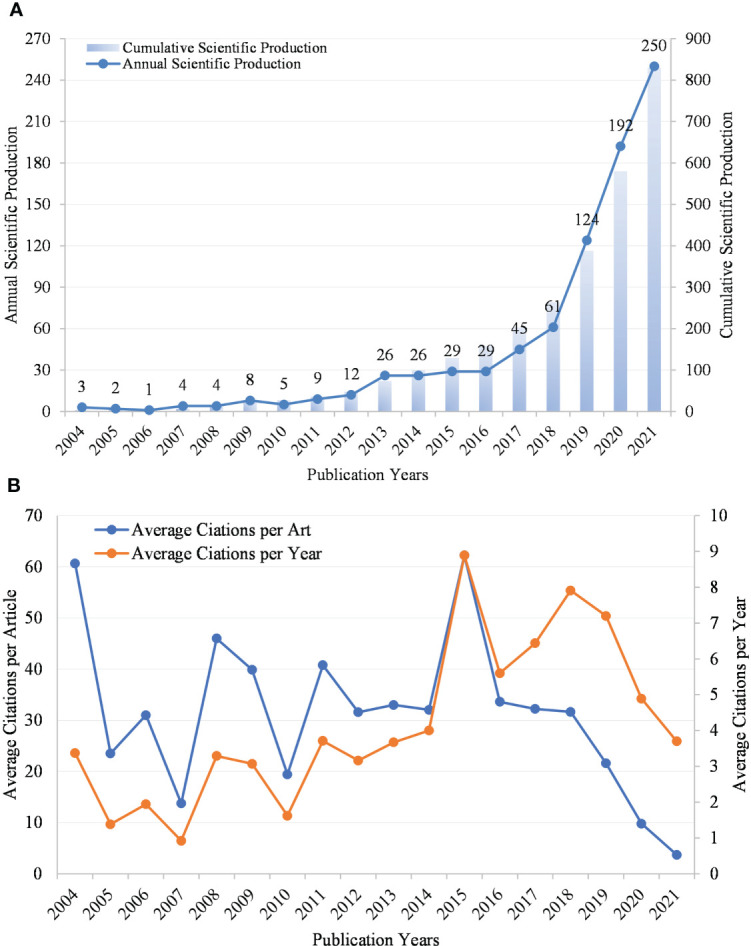
**(A)** Annual scientific production and cumulative scientific production of publications in TCM/GM. **(B)**The average citations per article and average citations of articles per year in TCM/GM.

**Figure 1A** shows the number of papers (Np) from 2004 to 2021 in TCM/GM research. The annual growth rate in Np was 29.71%, and the doubling time of the Np appeared in 2018. From 2004 to 2010, the annual Np and total Np (n=27, 3.25%) for this period were very small, the average annual Np was 2.14 and relative annual growth rate was 8.89%, indicating that TCM/GM research was in its infancy. The annual Np and total Np (n=176, 21.2%) in this period increased slowly from 2011 to 2017, the average annual Np was 32.14 and relative annual growth rate was 30.77%. From 2018 to 2021, the annual Np and total Np (n=627, 75.5%) for this period increased rapidly, the average annual Np reached 180.75 and relative annual growth rate was 60.03%.

**Figure 1B** shows the average citations per article and the average citations per year. In 2004 and 2019, the average citations per article were rather high (but the Np was small), indicating that some papers in these years may have important guiding and pioneering significance. In 2015 and 2018, the average citations per year were rather high, indicating that some papers in these years had significant relevance. From 2004 to 2018, the number of citations per year showed an upward trend. After 2018, the average citations per year showed a downward trend, which is exactly the opposite of the annual Np, which is considered likely to be associated with fewer citations for the latest publications.

### 3.2 Characteristics of papers in TCM/GM

#### 3.2.1 Main authors

**Table 2** lists the top 10 authors and their H-index and TC, of which Duan Jin-ao (n = 24), Jiang Shu (n = 17), Qian Da-wei (n = 16), Shang Er-xin (n = 16), Li, Xiao-bo (n = 15) ranked the top five in the Np. Duan Jin-ao from Nanjing University of Chinese Medicine had the most Np and H-index, and Tong Xiao-lin from China Academy of Chinese Medical Sciences had the most TC, indicating that their papers were of high quality and had a great impact on TCM/GM research. Notably, half of the authors were from Nanjing University of Chinese Medicine. Moreover, there were two authors from South Korea.

**Table 2:** The top 10 productive authors in the TCM/GM.

Rank	Author (Full Names)	Np	TC	H-index	Affiliations	Countries
1	Duan, Jin-ao	24	475	13	Nanjing University of Chinese Medicine	China
2	Jiang, Shu	17	350	10	Nanjing University of Chinese Medicine	China
3	Qian, Da-wei	16	328	10	Nanjing University of Chinese Medicine	China
4	Shang, Er-xin	16	338	10	Nanjing University of Chinese Medicine	China
5	Li, Xiao-bo	15	220	8	Shanghai Jiao Tong University	China
6	Guo, Jian-ming	14	199	9	Nanjing University of Chinese Medicine	China
7	Peng, Ying	14	204	7	Shanghai Jiao Tong University	China
8	Tong, Xiao-lin	12	703	9	China Academy of Chinese Medical Sciences	China
9	Kim, Dong-hyun	12	341	10	Kyung Hee University	South Korea
10	Kim, Hojun	11	297	8	Dongguk University	South Korea

**Figure 2:**
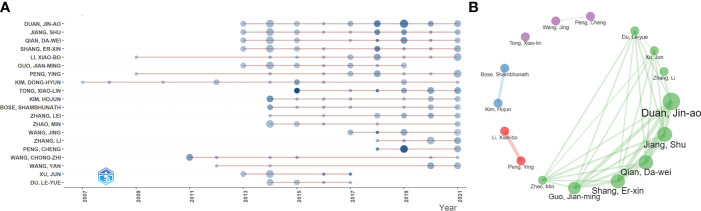
**(A)** The top 20 authors’ annual publications over time in TCM/GM (the size of the circle represents the number of publications, and the larger the circle, the more the number of publications; the depth of the circle represents the average annual citation, and the darker the color, the more citations). **(B)** The top 20 authors’ co-authorship network (remove isolated nodes) in TCM/GM (each node represents an author, the size of the node represents the number of published articles, the line represents the collaboration network between authors, and the thickness of the line represents the strength of collaboration).

**Figure 2A** shows the annual scientific productivity of the top 20 authors. Most authors had published TCM/GM-related papers since 2014, and most authors’ influential papers were published in 2018 and 2019 (the darkest in the graph). **Figure 2B** shows the collaborations of the top 20 authors. Among them, the cooperation group with Duan Jin-ao as the core had the most collaborators, and most of the collaborators belong to the same institution (Nanjing University of Chinese Medicine), showing the tendency for cooperation within the institution.

#### 3.2.2 Major countries/regions and institutions

**Figure 3:**
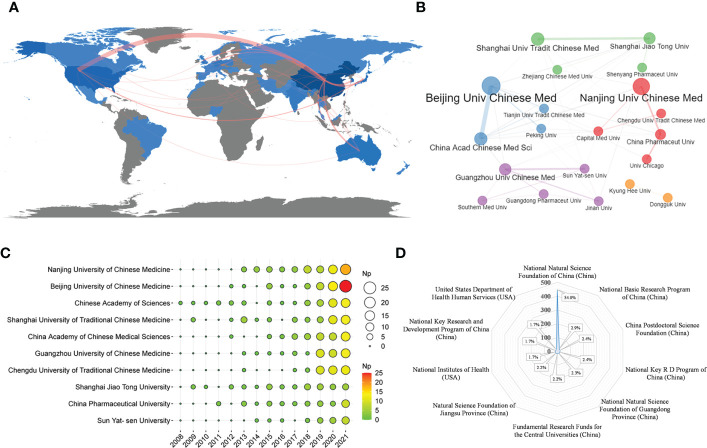
**(A)** Country scientific production and international collaboration network in TCM/GM (the red line represents the collaboration network between countries, the thickness of the line represents the strength of collaboration). **(B)** Collaboration network of the top 20 corresponding institutions in TCM/GM. **(C)** Annual scientific production of the top 10 most productive institutions over time in TCM/CM based on WoS (the size of the circle represents the number of publications, and the larger the circle, the more the number of publications). **(D)** The top 10 funding agencies in the support of TCM/GM research.

**Figure 3A** shows the country distribution of papers. The papers were mainly from China (716), accounting for about 86.27% of total output, followed by USA (n = 58), South Korea (n = 44), Japan (n = 26) and Australia (n = 12) (**Table 3**). More than 800 institutions were involved in this study, and the top 10 most productive institutions were shown in **Table 3**, of which Nanjing University of Chinese Medicine (n = 63), Beijing University of Chinese Medicine (n = 59), Chinese Academy of Sciences (n = 47), Shanghai University of Traditional Chinese Medicine (n = 46) and China Academy of Chinese Medical Sciences (n = 43) were among the top five. The top 10 institutions all were from China, of which Chinese Academy of Chinese Medical Sciences had the most TC and Nanjing University of Chinese Medicine had the highest H-index.

**Table 3:** The top 10 productive countries/regions and institutions involved in the TCM/GM.

Rank	Countries	Np	TC	H-index	Institutions	Np	TC	H-index
1	China	716	12325	48	Nanjing University of Chinese Medicine (China)	63	1178	19
2	USA	58	2598	24	Beijing University of Chinese Medicine (China)	59	719	15
3	South Korea	44	886	16	Chinese Academy of Sciences (China)	47	949	16
4	Japan	26	503	14	Shanghai University of Traditional Chinese Medicine (China)	46	719	16
5	Australia	12	234	6	China Academy of Chinese Medical Sciences (China)	43	1259	17
6	Germany	7	192	6	Guangzhou University of Chinese Medicine (China)	39	581	14
7	India	5	83	4	Chengdu University of Traditional Chinese Medicine (China)	37	509	11
8	UK	4	111	4	Shanghai Jiao Tong University (China)	35	1099	17
9	Netherlands	4	33	3	China Pharmaceutical University (China)	32	602	13
10	Poland	4	112	4	Sun Yat-sen University (China)	22	307	11

**Figure 3B** depicts the partnership of the top 20 institutions in TCM/GM. Among them, Beijing University of Chinese Medicine and China Academy of Chinese Medical Sciences had the closest partnership. **Figure 3C** shows the annual scientific productivity of the top 10 institutions. We can see that Beijing University of Chinese Medicine had the most Np in the past two years. **Figure 3D** depicts the main funding agencies, which mainly were from China, especially the National Natural Science Foundation of China (accounting for 54%), indicating that China has strong support for research in related fields.

#### 3.2.3 Main journals

**Figure 4:**
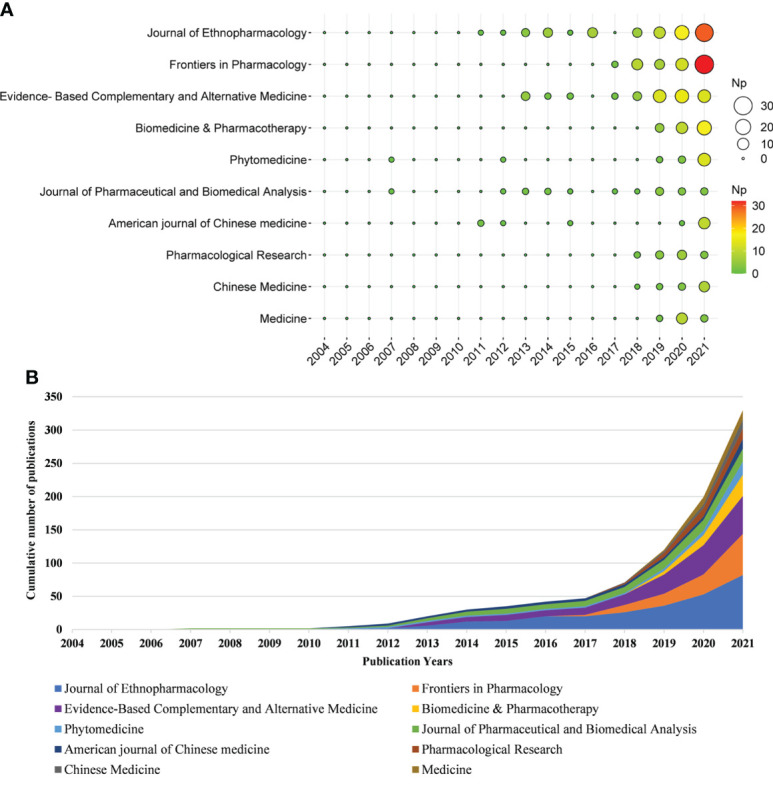
**(A)** The top 10 journals’ annual publications over time in TCM/GM (the size of the circle represents the number of papers, and the larger the circle, the more the number of papers). **(B)** The cumulative number of papers of the top 10 journals in TCM/GM.

These papers were published in 241 journals. **Table 4** shows the top 10 journals in the Np, of which *Journal of Ethnopharmacology* had the most Np (n = 82), followed by *Frontiers in Pharmacology* (n = 62), *Evidence-Based Complementary and Alternative Medicine* (n = 57) and *Biomedicine & Pharmacotherapy* (n = 32). The TC can show the importance of the journal, and the H-Index can evaluate the academic influence of journals. In the top 10 most productive journals, the *Journal of Ethnopharmacology* had the highest TC and H-index, followed by *Frontiers in Pharmacology*, and the *Journal of Pharmaceutical and Biomedical Analysis*. **Figure 4** summarizes the annual Np and the cumulative Np in the top 10 journals. The cumulative Np in these journals was 376, accounting for about 37.94% of all papers, indicating that their excellent productivity.

**Table 4:** The top 10 productive journals in the TCM/GM.

Rank	Journals	Np	TC	H-index	IF	Partition	Countries
1	Journal of Ethnopharmacology	82	1499	21	5.195	Q2	Ireland
2	Frontiers in Pharmacology	62	1101	16	5.988	Q1	Switzerland
3	Evidence-Based Complementary and Alternative Medicine	57	396	11	2.650	Q3	UK
4	Biomedicine & Pharmacotherapy	32	383	11	7.419	Q1	France
5	Phytomedicine	20	149	7	6.656	Q1	Germany
6	Journal of Pharmaceutical and Biomedical Analysis	19	349	13	3.571	Q2	Netherlands
7	American journal of Chinese medicine	15	298	6	6.005	Q1	USA
8	Pharmacological Research	15	523	12	10.334	Q1	UK
9	Chinese Medicine	14	93	6	4.546	Q1	UK
10	Medicine	14	22	3	1.817	Q3	USA

### 3.3 Analysis of cited papers in TCM/GM

#### 3.3.1 Historical cited papers of TCM/GM research

**Figure 5:**
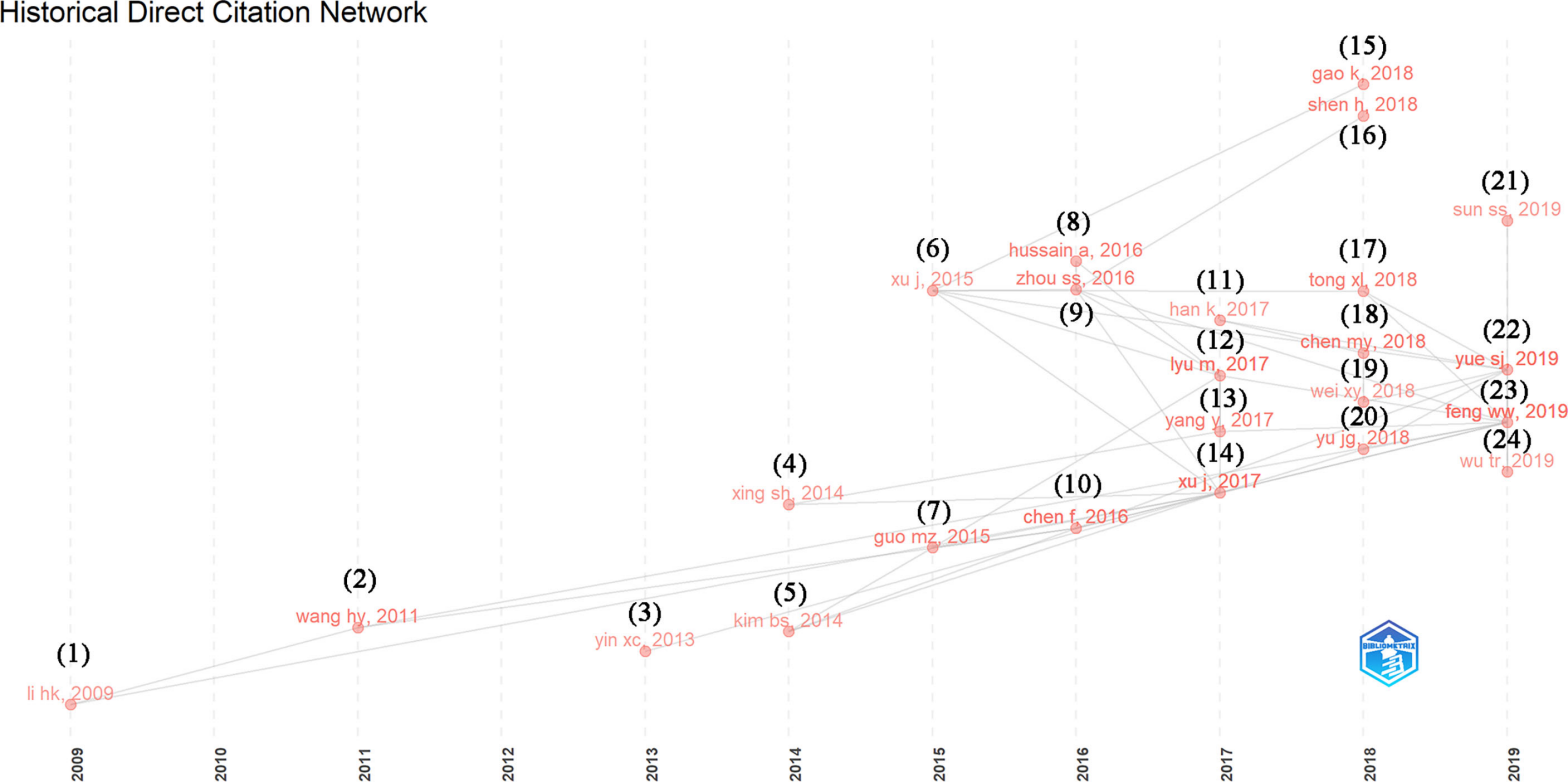
Historical direct citation network in TCM/GM (each paper is represented by the first author and year, grey lines between points indicate citation relationship, and the papers corresponding to each serial number are shown in **Table 7**).

Historical direct citation analysis can quickly identify the most relevant and cited significant papers in the development process of a field, draw a historical direct citation map according to the time series, and then trace the source year by year to analyze the historical development. **Figure 5** shows the citation relationship of several classic papers, which appeared from 2009 to 2019. Two indicators, local citation score (LCS) and global citation score (GCS), were used to examine the research importance of the classic papers. LCS corresponds to the citations of a paper in the downloaded dataset, and GCS represents the times a paper had been cited by all papers in the WoS database (**Table 5**).

**Table 5:** The papers of historical direct citation network in the TCM/GM.

No.	Title	Document Type	First Author	Journals	Year	LCS	GCS
1	Traditional Chinese Medicine: Balancing the Gut Ecosystem	Review	Li, HK	Phytother. Res.	2009	14	42
2	Bioactivity Enhancement of Herbal Supplements by Intestinal Microbiota Focusing on Ginsenosides	Review	Wang, HY	Am. J. Chin. Med.	2011	11	82
3	Structural changes of gut microbiota in a rat non-alcoholic fatty liver disease model treated with a Chinese herbal formula	Article	Yin, XC	Syst. Appl. Microbiol.	2013	12	58
4	Simulated gastrointestinal tract metabolism and pharmacological activities of water extract of Scutellaria baicalensis roots	Article	Xing, SH	J. Ethnopharmacol.	2014	10	46
5	The anti-obesity effect of Ephedra sinica through modulation of gut microbiota in obese Korean women	Article	Kim, BS	J. Ethnopharmacol.	2014	12	61
6	Structural modulation of gut microbiota during alleviation of type 2 diabetes with a Chinese herbal formula	Article	Xu, J	ISME J.	2015	52	275
7	Red Ginseng and Semen Coicis can improve the structure of gut microbiota and relieve the symptoms of ulcerative colitis	Article	Guo, MZ	J. Ethnopharmacol.	2015	20	65
8	Daesiho-Tang Is an Effective Herbal Formulation in Attenuation of Obesity in Mice through Alteration of Gene Expression and Modulation of Intestinal Microbiota	Article	Hussain, A	PLoS One	2016	12	37
9	Gut microbiota-involved mechanisms in enhancing systemic exposure of ginsenosides by coexisting polysaccharides in ginseng decoction	Article	Zhou, SS	Sci Rep	2016	26	122
10	Could the gut microbiota reconcile the oral bioavailability conundrum of traditional herbs?	Review	Chen, F	J. Ethnopharmacol.	2016	22	100
11	*In vivo* therapeutic effect of combination treatment with metformin and Scutellaria baicalensis on maintaining bile acid homeostasis	Article	Han, K	PLoS One	2017	10	27
12	Balancing Herbal Medicine and Functional Food for Prevention and Treatment of Cardiometabolic Diseases through Modulating Gut Microbiota	Article	Lyu, M	Front. Microbiol.	2017	12	100
13	Gut microbiota drives the attenuation of dextran sulphate sodium-induced colitis by Huangqin decoction	Article	Yang, Y	Oncotarget	2017	16	61
14	Understanding the Molecular Mechanisms of the Interplay Between Herbal Medicines and Gut Microbiota	Article	Xu, J	Med. Res. Rev.	2017	48	149
15	Effects of Qijian mixture on type 2 diabetes assessed by metabonomics, gut microbiota and network pharmacology	Article	Gao, K	Pharmacol. Res.	2018	11	54
16	Ginseng polysaccharides enhanced ginsenoside Rb1 and microbial metabolites exposure through enhancing intestinal absorption and affecting gut microbial metabolism	Article	Shen, H	J. Ethnopharmacol.	2018	10	44
17	Structural Alteration of Gut Microbiota during the Amelioration of Human Type 2 Diabetes with Hyperlipidemia by Metformin and a Traditional Chinese Herbal Formula: a Multicenter, Randomized, Open Label Clinical Trial	Article	Tong, XL	mBio	2018	32	146
18	Huang-Lian-Jie-Du-Decoction Ameliorates Hyperglycemia and Insulin Resistant in Association With Gut Microbiota Modulation	Article	Chen, MY	Front. Microbiol.	2018	16	60
19	Xiexin Tang improves the symptom of type 2 diabetic rats by modulation of the gut microbiota	Article	Wei, XY	Sci Rep	2018	30	110
20	Gancao-Gansui combination impacts gut microbiota diversity and related metabolic functions	Article	Yu, JG	J. Ethnopharmacol.	2018	12	22
21	An insoluble polysaccharide from the sclerotium of Poria cocos improves hyperglycemia, hyperlipidemia and hepatic steatosis in ob/ob mice *via* modulation of gut microbiota	Article	Sun, SS	Chin. J. Nat. Med.	2019	13	74
22	Gut microbiota modulation with traditional Chinese medicine: A system biology-driven approach	Review	Yue, SJ	Pharmacol. Res.	2019	11	28
23	Gut microbiota, a new frontier to understand traditional Chinese medicines	Review	Feng, WW	Pharmacol. Res.	2019	32	133
24	Gut commensal Parabacteroides goldsteinii plays a predominant role in the anti-obesity effects of polysaccharides isolated from Hirsutella sinensis	Article	Wu, TR	Gut	2019	26	292

In 2009, a review paper titled “*Traditional Chinese Medicine: Balancing the Gut Ecosystem*” proposed that TCM plays an important role in restoring the gut ecological balance, and the multi-channel regulation of the human gut ecosystem may be a large part of the efficacy during TCM treatment (Li et al., 2009). In 2011, a review article showed that GM can induce comprehensive metabolism of herbal components and enhance the biological activity of ginsenosides (Wang et al., 2011). In 2013, a study showed that Chinese herbal formula can improve the GM in rats with non-alcoholic fatty liver disease (NAFLD) and return it to normal levels (Yin et al., 2013). In 2014, a clinical study showed that Ephedra sinica can exert an anti-obesity effect by regulating the GM of obese women (Kim et al., 2014). An *in vitro* study demonstrated that the presence of GM plays an important role in the gastrointestinal metabolism of the water extract of root of Scutellaria baicalensis (Xing et al., 2014). In 2015, an experimental study showed that Red Ginseng and Semen Coicis can improve the structure of GM, promote the growth of probiotics such as Bifidobacterium and Lactobacillus, and relieve the symptoms of ulcerative colitis (UC) (Guo et al., 2015). A clinical study showed that structure changes of GM (an increase in beneficial bacteria) induced by Gegen Qinlian Decoction (GQD) were associated with its anti-diabetic effect (Xu et al., 2015), which provided an important reference for TCM microecology research. In 2016, A review article on herb-microbiota interactions showed that TCM can play a role in promoting health and preventing diseases by affecting the structure of GM, and some herbal components can play their therapeutic roles through the GM-mediated biotransformation (Chen et al., 2016). Zhou et al. (2016) found that Ginseng polysaccharides can improve intestinal metabolism and absorption of ginsenosides, reinstate the disturbed GM, and promote the growth of *Lactobacillus* and *Bacteroides*. This study showed that even TCM polysaccharides that cannot be digested by the host can still indirectly promote the therapeutic effect, which endows TCM polysaccharides with new effects. Hussain et al. (2016) showed that Daesiho-tang reduced obesity in high-fat diet (HFD)-fed mice by altering gene expression and modulating GM. In 2017, a review article provided an overview of the molecular mechanisms underlying the interaction between TCM and GM (Xu et al., 2017). The other review article summarized herbal and functional foods for the prevention and treatment of cardiometabolic diseases by modulating GM and exerting prebiotic-like activities (Lyu et al., 2017). A study confirmed that Huangqin Decoction can improve dextran sulphate sodium (DSS)-induced colitis by altering the GM (Yang et al., 2017). An animal study demonstrated that the synergistic effect of metformin and Scutellaria baicalensis in lowering cholesterol levels by fecal excretion of bile acids (Han et al., 2017).

In 2018, an animal experiment showed Huang-Lian-Jie-Du Decoction (HLJDD) could improve hyperglycemia and restore the structure and function of dysregulated GM to a normal state by increasing short-chain fatty acids (SCFAs)-producing bacteria and reducing pathogenic bacteria in type 2 diabetes mellitus (T_2_DM) rats, which provides new ideas for the study of the mechanism of TCM in the treatment of T_2_DM (Chen et al., 2018). Gao et al. (2018) found that Qijian Mixture can effectively alleviate T_2_DM, and this effect was related to the altered characteristics of metabolite profiles and GM. Wei et al. (2018) showed that Xiexin-Tang could significantly improve hyperglycemia, lipid metabolism dysfunction and inflammation in T_2_DM rats, and some GM were closely related to T_2_DM-related indicators. Subsequently, a clinical trial (Tong et al., 2018) showed that the TCM compound AMC can reduce the hyperlipidemia in patients with diabetes by changing the structure and diversity of the GM and regulating the probiotics of GM. A further animal experiment (Shen et al., 2018) demonstrated that Ginseng polysaccharides can alleviate DSS-induced colitis and enhance the systemic exposure of Rb1 by enhancing microbial deglycosylation and intestinal epithelial uptake of Rb1. The theory of “eighteen incompatible medicaments” in TCM is the most representative case of herbal-herbal interactions. Gancao and Gansui are one of the incompatible herbal pairs. Yu et al. (2018) showed that the Gancao-Gansui combination did not exacerbate gastrointestinal tissue or functional damage, but caused GM dysbiosis and increased the abundance of some rare genera such as *Desulfovibrio* and *Mycoplasma*. In 2019, a study (Wu et al., 2019) revealed the Parabacteroides goldsteinii plays a major role in the anti-obesity effect of polysaccharides isolated from Hirsutella sinensis. The other study (Sun et al., 2019) showed that an insoluble polysaccharide in sclerotia of Poria cocos can ameliorate hyperglycemia, hyperlipidemia and hepatic steatosis in mice by modulating GM. Two reviews (Feng et al., 2019; Yue et al., 2019) summarized the interactions between TCM and GM, including its theory, mechanism, and the future prospects and challenges of GM in TCM.

#### 3.3.2 Top 20 most cited original research articles in TCM/GM

Highly cited papers are one of the most valuable indicators in bibliometric methods, which usually are highly recognized. **Table 6** lists the top 20 most cited papers in original research. Some papers have been outlined above, for instance, TCM can improve diabetes and insulin resistance (Xu et al., 2015; Liu et al., 2018; Tong et al., 2018; Wei et al., 2018), obesity (Kim et al., 2014; Wu et al., 2019), colitis (Guo et al., 2015; Yang et al., 2017) by adjusting the GM, here we analyze some other highly cited papers.

**Table 6:** The top 20 cited original research articles related to the TCM/GM.

Rank	Title	First author	Year	Journals	IF	TC
1	Ganoderma lucidum reduces obesity in mice by modulating the composition of the gut microbiota	Chang, CJ	2015	Nat. Commun.	17.694	680
2	Gut commensal Parabacteroides goldsteinii plays a predominant role in the anti-obesity effects of polysaccharides isolated from Hirsutella sinensis	Wu, TR	2019	Gut	31.793	292
3	Structural modulation of gut microbiota during alleviation of type 2 diabetes with a Chinese herbal formula	Xu, J	2015	ISME J.	11.217	275
4	Polyphenol-rich extract of pomegranate peel alleviates tissue inflammation and hypercholesterolaemia in high-fat diet-induced obese mice: potential implication of the gut microbiota	Neyrinck, AM	2013	Br. J. Nutr.	4.125	158
5	Structural Alteration of Gut Microbiota during the Amelioration of Human Type 2 Diabetes with Hyperlipidemia by Metformin and a Traditional Chinese Herbal Formula: a Multicenter, Randomized, Open Label Clinical Trial	Tong, XL	2018	mBio	7.786	146
6	Gut microbiota-involved mechanisms in enhancing systemic exposure of ginsenosides by coexisting polysaccharides in ginseng decoction	Zhou, SS	2016	Sci Rep	4.996	122
7	Xiexin Tang improves the symptom of type 2 diabetic rats by modulation of the gut microbiota	Wei, XY	2018	Sci Rep	4.996	110
8	Prebiotic Effect of Fructooligosaccharides from Morinda officinalis on Alzheimer’s Disease in Rodent Models by Targeting the Microbiota-Gut-Brain Axis	Chen, DL	2017	Front. Aging Neurosci.	5.702	96
9	Phytonutrient diet supplementation promotes beneficial Clostridia species and intestinal mucus secretion resulting in protection against enteric infection	Wlodarska, M	2015	Sci Rep	4.996	86
10	The effects of co-administration of probiotics with herbal medicine on obesity, metabolic endotoxemia and dysbiosis: A randomized double-blind controlled clinical trial	Lee, SJ	2014	Clin. Nutr.	7.643	83
11	Structural Changes of Gut Microbiota during Berberine-Mediated Prevention of Obesity and Insulin Resistance in High-Fat Diet-Fed Rats	Zhang, X	2012	PLoS One	3.752	80
12	An insoluble polysaccharide from the sclerotium of Poria cocos improves hyperglycemia, hyperlipidemia and hepatic steatosis in ob/ob mice *via* modulation of gut microbiota	Sun, SS	2019	Chin. J. Nat. Med.	3.887	74
13	Role of human gut microbiota metabolism in the anti-inflammatory effect of traditionally used ellagitannin-rich plant materials	Piwowarski, JP	2014	J. Ethnopharmacol.	5.195	68
14	Red Ginseng and Semen Coicis can improve the structure of gut microbiota and relieve the symptoms of ulcerative colitis	Guo, MZ	2015	J. Ethnopharmacol.	5.195	65
15	Colon cancer chemopreventive effects of baicalein, an active enteric microbiome metabolite from baicalin	Wang, CZ	2015	Int. J. Oncol.	5.884	63
16	Metabolism of Rutin and Poncirin by Human Intestinal Microbiota and Cloning of Their Metabolizing alpha-L-Rhamnosidase from Bifidobacterium dentium	Bang, SH	2015	J. Microbiol. Biotechnol.	3.277	63
17	Gut microbiota drives the attenuation of dextran sulphate sodium-induced colitis by Huangqin decoction	Yang, Y	2017	Oncotarget	—	61
18	The anti-obesity effect of Ephedra sinica through modulation of gut microbiota in obese Korean women	Kim, BS	2014	J. Ethnopharmacol.	5.195	61
19	Mushroom polysaccharides from Ganoderma lucidum and Poria cocos reveal prebiotic functions	Khan, I	2018	J. Funct. Food.	5.223	59
20	Berberine Modulates Gut Microbiota and Reduces Insulin Resistance *via* the TLR4 Signaling Pathway	Liu, D	2018	Exp. Clin. Endocrinol. Diabet.	2.426	58

Highly cited papers mainly focused on the effect of TCM on GM to improve obesity and obesity-related diseases. A 2015 animal study (Chang et al., 2015) found Ganoderma lucidum could reduce obesity in mice by modulating GM, which had the highest TC. It pointed out a new direction for studying the mechanism of TCM, and has important guiding significance for TCM microecology research. Furthermore, oral administration of pomegranate peel extract can alleviate tissue inflammation and hypercholesterolemia in HFD-induced obese mice by modulating GM and promoting the growth of *Bifidobacteria* (Neyrinck et al., 2013). The effect of berberine on preventing obesity and insulin resistance in HFD-fed rats is partially mediated by the GM, which may reduce the exogenous antigen load of the host and increase the SCFAs level in the gut (Zhang et al., 2012). An insoluble polysaccharide from the sclerotium of Poria cocos can improve hyperglycemia, hyperlipidemia and hepatic steatosis by regulating GM (Sun et al., 2019). Fructooligosaccharides from Morinda officinalis can exert prebiotic effects on animal models of Alzheimer’s disease by targeting the microbiota-gut-brain axis (Chen et al., 2017). Moreover, probiotics combined with TCM had synergistic modulatory effects on GM, and TCM seems to act as a potential substitute for probiotics. A clinical trial (Lee et al., 2014) showed that co-administration of probiotics with herbal remedies had an effect on the GM in obese patients, resulting in significant reductions in body weight and waist circumference, which was important for the exploration of new prebiotics.

Several articles mentioned the relationship between GM and TCM in other aspects. For example, eugenol may enhance the mucosal barrier by increasing the thickness of the inner mucus layer through microbial stimulation, thereby preventing invading pathogens and diseases (Wlodarska et al., 2015). The metabolism of human GM plays an important role in the anti-inflammatory effect of traditional tannin-rich plant materials (Piwowarski et al., 2014). GM can play an important role in chemical prevention of colon cancer in Scutellaria baicalensis (Wang et al., 2015). Mushroom polysaccharides from Ganoderma lucidum and Poria cocos act as prebiotics to regulate GM composition, thus potentially contributing to the health promotion effect (Khan et al., 2018). Human GM has an impact on the metabolism and transformation of natural products, such as a study showing that intestinal bacteria play an important role in the metabolism and pharmacological effects of rhamnoglycosides (Bang et al., 2015).

#### 3.3.3 Most local cited references and analysis of burst references

**Figure 6:**
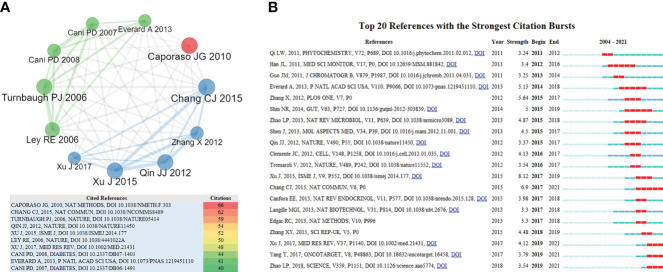
**(A)** The most cited references and citations (frequency ≥ 40). **(B)** The top 20 references with the most bursts (The years between ‘start’ and ‘end’ represent the period in which references are more influential. The years with light green indicate that the references have not yet appeared, the years with dark green indicate that the references have less influence, and the years with red indicate that the references have more influence).

**Figure 6A** shows the references with the most citations. In 2006, a mouse study (Turnbaugh et al., 2006) identified the GM as a contributing factor in the pathophysiology of obesity, which showed that obesity-associated GM had enhanced and transmissible ability to obtain energy from diet, and compared to ‘lean microbiota’, colonization of ‘obese microbiota’ in germ-free mice can result in a significantly greater increase in body fat. A clinical study (Ley et al., 2006) showed that the relative proportion of Bacteroides was reduced in obese people compared to lean people, and that this proportion increased with weight loss on two types of low-calorie diet. In 2010, Caporaso JG et al. (Caporaso et al., 2010) developed QIIME, which allows analysis of high-throughput community sequencing data. A 2012 clinical metagenome study (Qin et al., 2012) of the GM in T_2_DM suggested that gut microbial markers may help to classify T_2_DM. In 2015, Chang et al. (2015) verified Ganoderma lucidum could reduce obesity in mice by modulating the composition of GM. Xu et al. (2015) found that GQD could enrich the population of beneficial bacteria, such as Faecalibacterium, which is associated with its anti-diabetic effect. In 2017, a review article (Xu et al., 2017) provided a detailed description of the molecular mechanisms underlying the interactions between TCM and GM.

**Figure 6B** shows the top 20 references with the most bursts in this study. As shown in **Figure 6B**, most of the valuable burst references were from *Nature* and its sub-journals, showing the important inspiration of high-impact journals for TCM/GM research. In 2011, A review article (Han et al., 2011) mentioned that modulating GM may act as an anti-diabetic mechanism of berberine, providing a new explanation for the therapeutic effects of berberine. In 2012, two papers in *Nature* suggested that patients with T_2_DM were characterized by the GM dysbiosis, a decrease in the abundance of some commonly butyrate-producing bacteria, and an increase in various opportunistic pathogens (Qin et al., 2012); there are functional interactions between the GM and host metabolism (Tremaroli and Bäckhed, 2012). A review article (Clemente et al., 2012) in *Cell* detailed the important role of human GM in host health and specified changes of GM associated with disease. In 2013, a study (Everard et al., 2013) showed that the abundance of *Akkermansia muciniphila* was reduced in obese and T_2_DM mice, but prebiotic feeding normalized its abundance, which was associated with an improved metabolic profile. Two reviews (Shen et al., 2013; Zhao, 2013)explored the interrelationships between the GM, obesity and insulin resistance. In 2014, an article (Shin et al., 2014) showed that metformin treatment can increase *Akkermansia*, which may be associated with improved glucose homeostasis in diet-induced obese mice. Diet can rapidly and reproducibly alter the GM (David et al., 2014). In 2015, a study (Canfora et al., 2015) showed that GM metabolites SCFAs played an important role in control of body weight and insulin sensitivity. An experimental study (Feng et al., 2015) revealed that the GM can convert berberine into a form that can be absorbed by the gut. In 2018, a clinical study in *Science* suggested that dietary fiber can selectively boost GM to alleviate T_2_DM. It can be seen that most of the papers come from the research on GM in modern medicine, especially the correlation study between GM and obesity, indicating that modern medicine has important enlightening significance for TCM research.

### 3.4 Analysis of keywords in TCM/GM

#### 3.4.1 High-frequency keywords and burst keywords

**Figure 7:**
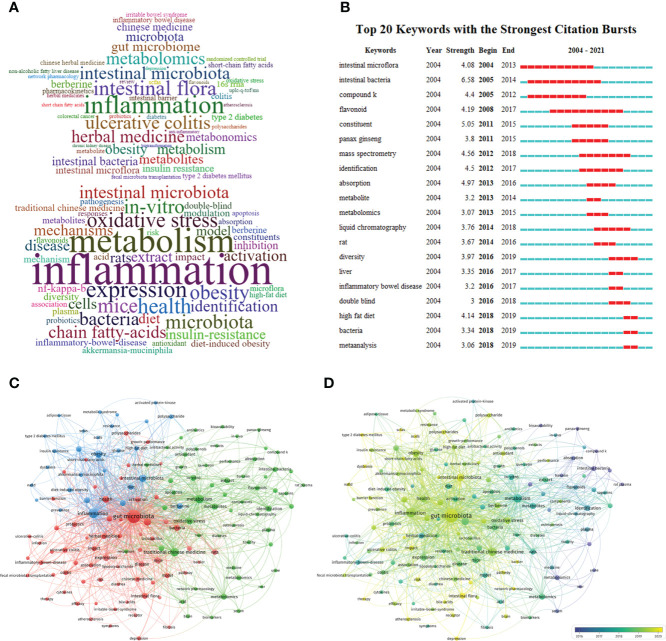
**(A)** Distribution of top 50 keywords in TCM/GM (exclude main search terms, the upper part is the author keywords, and the lower part is the keywords plus). **(B)** The top 20 keywords with the most bursts base on CiteSpace (The year between ‘start’ and ‘end’ represents the period in which keywords are more influential. The year with light green indicates that the keywords have not yet appeared, the year with dark green indicates that the keywords have less influence, and the year with red indicates that the keywords have more influence). **(C)** Cluster analysis of high-frequency keywords (frequency ≥ 10) in TCM/GM (different colors represent different clusters, the size of the circle represents the frequency the keywords appear, and the thickness of the line represents the total link strength between keywords). **(D)** Trends in keywords (frequency ≥ 10) over time base on VOSviewer (the blue dots represent the earliest keywords and the yellow dots represent the latest keywords).

A total of 4049 keywords (1951 author’s keywords and 2118 keywords plus) were extracted from the imported papers. **Figure 7A** depicts the author’s keywords and keywords plus with the top 50 frequencies. Among author’s keywords, the most used keywords (exclude search terms) were “inflammation”, “ulcerative colitis”, “obesity”, “berberine”, “insulin resistance”, “type 2 diabetes”, “inflammatory bowel disease”, “irritable bowel syndrome”, “non-alcoholic fatty liver disease”, “metabolomics”, “metabolism”, “metabolites”, “pharmacokinetics”, “short-chain fatty acids”, “oxidative stress”, etc. Among keywords plus, the most used keywords (exclude search terms) were “inflammation”, “metabolism”, “metabolomics”, “expression”, “health”, “oxidative stress”, “obesity”, “chain fatty-acids”, “mechanisms”, “insulin-resistance”, “inflammatory bowel disease”, “antioxidant”, “flavonoids”, etc.

Through keywords with the most bursts detection, we can understand the development and changes of research hotspots, trends, and frontier dynamics within a certain period of time. A total of 20 burst keywords in this study were obtained, as shown in **Figure 7B**. The result showed that in the early days (before 2016), the effect of GM on the metabolism and biotransformation of TCM ingredients especially panax ginseng compounds K (one of the metabolites of ginsengoside) and flavonoids was the main focus. In the past few years (after 2016), the research focus was mainly on the effects of TCM on the GM of HFD-induced animal models, and the mechanism and clinical research of regulating GM imbalance to prevent and treat HFD-induced glycolipid metabolism diseases and inflammatory bowel disease (IBD). TCM/GM research had gone through the stage from pharmaceutical research to preclinical research, and finally to clinical research.

#### 3.4.2 Cluster and trends analysis of high-frequency keywords

Based on the frequency of two or more keywords appearing at the same time, clustering analysis is carried out. This study treated each clustered keyword as a category, then merged it into the cluster with the highest similarity, finally classified all individuals into a category base on same color. **Figure 7C** shows the clustering results in TCM/GM, which can be divided into three categories.

##### Cluster 1 (blue topic)

This category was mainly about the interactions between TCM and GM plays an important role in glycolipid metabolic diseases and explains the mechanism of interplay. (Keywords: inflammation, obesity, berberine, mechanisms, insulin-resistance, diet-induced obesity, high fat diet, fatty liver disease, type 2 diabetes mellitus, metabolic syndrome, polysaccharide).

##### Cluster 2 (red topic)

This category was mainly about the relationship between GM, TCM and lower gastrointestinal diseases. (Keywords: fecal microbiota, herbal medicine, probiotics, inflammatory-bowel-disease, ulcerative-colitis, irritable-bowel-syndrome, efficacy, association, mechanisms, short-chain fatty acids, pathogenesis, expression, health, disease, bile-acids, barrier function).

##### Cluster 3 (green theme)

This category is mainly related to metabolism of TCM and GM-mediated metabolite, as well as GM-mediated pharmacokinetics and biotransformation of TCM. (Keywords: oxidative stress, antioxidant, identification, flavonoids, pharmacokinetics, biotransformation, liquid chromatography, mass spectrometry, panax ginseng, apoptosis).

Overlaid visual maps, similar to concurrency graphs, are an effective way to predict future trends and hotspots. VOSviewer uses different colors for each keyword in the images based on the average time they appear in all included publications, as shown in **Figure 7D**, where the blue circles represent the earliest keywords and the yellow circles represent keywords appear in recent years. From 2016 to 2020, there are relatively unbalanced development dynamics in three clusters, more yellow nodes are in the cluster 1 and 2. This showed that clinical research has become a research hotspot in recent years.

## 4 Discussion

TCM has been widely used worldwide as a complementary alternative therapy because of its clinical efficacy and reduced side effects for thousands of years, but the mechanisms by which TCM prevents and treats disease remains unclear (Xu et al., 2017). In recent years, with the rapid development of high-throughput sequencing, 16S rRNA detection technology and bioinformatics methodology (Caporaso et al., 2010; Qin et al., 2012), people have gradually realized that GM plays an important role in the TCM treatment of disease (Lin et al., 2019; Zheng et al., 2020). Along with this, how TCM regulates the GM to achieve the effect of curing diseases has also become a hot topic that needs to be solved. To better understand the interactions between GM and TCM, we reviewed and analyzed related research papers and summarized the influence of GM on TCM by means of bibliometric methods, and discussed the mechanism of the interactions between the GM and TCM, and provides information for the development, utilization and modernization of TCM.

### 4.1 Current status and characteristics of literature

The number of publications in TCM/GM can reflect the development stage it had experienced. From the annual Np, 2004-2010 belonged to the initial stage of TCM/GM research. During this period, the GM research had just started, such as the official launch of the Human Microbiome Project and the China-France human intestinal metagenome research cooperation program in 2007. 2011-2017 was in a steady and slow development stage, which showed a consistent trend with the development of GM research (Yuan et al., 2021). And 2018 to present was in a stage of rapid development, which may be due to the rapid development of GM research and modernization of TCM, as well as the increasing interest of researchers in TCM/GM.

The study showed that the papers related to TCM/GM mainly came from China, followed by the US, South Korea and Japan. The top 10 institutions were all located in China, demonstrating good scientific research capabilities of China in TCM/GM. China had the most publications and was at the core of global cooperation, which can be related to long application history of TCM and the high attention and financial support of the government and community of China on the GM program such as funding and technical support of the National Natural Science Foundation and the Chinese Academy of Sciences Microbiome Program initiated in 2017. Nanjing University of Chinese Medicine and Beijing University of Chinese Medicine, as the top Chinese medicine universities in China, had published the most articles. Moreover, Nanjing University of Chinese Medicine published the most papers and had the highest H-index and most high-yield authors, showing its outstanding contribution to TCM/GM research.

The most productive author was Duan Jin-ao, who had contributed to the field of TCM/GM and was a leading pioneer in the study of the relationship between GM and TCM, especially the research of TCM metabolomics (Xiao et al., 2020; Zhang et al., 2021) and “eighteen incompatible medicaments” (Yu et al., 2018; Yu et al., 2019), and was at the core of the collaborative relationship between authors. Li Xiao-bo from Shanghai Jiao Tong University mainly focused on the GM of spleen deficiency syndrome and the intervention mechanism of Sijunzi decoction (Peng et al., 2020; Ma et al., 2021). Kim, Hojun from Dongguk University was mainly engaged in the research of TCM to improve obesity and metabolic syndrome by regulating GM (Kim et al., 2014; Hussain et al., 2016), while Kim, Dong-hyun from Kyung Hee University was busy studying the role of TCM metabolites in the treatment of diseases such as colitis (Kim et al., 2008; Jang et al., 2019). In terms of the impact of papers, Tong Xiao-lin from China Academy of Chinese Medical Sciences had the highest TC, who has long been engaged in clinical and scientific research on diabetes and its complications. Tong Xiao-lin published many highly cited papers (Xu et al., 2015; Tong et al., 2018) on diabetes, GM and TCM, his papers mainly focused on TCM/GM clinical research. Hsin-Chih Lai from Chang Gung University in Taiwan also had published many highly cited and influential papers (Chang et al., 2015; Wu et al., 2019) as the corresponding author. His papers mainly explored the relationship between GM and TCM such as Ganoderma lucidum and Hirsutella sinensis from the perspective of causality by fecal microbiota transplantation and antibiotic treatment rather than association in ordinary research, which has important inspiration for future research.

The study showed that the *Journal of Ethnopharmacology* had the most Np, the highest TC and H-index, which mainly focuses on the biological activities of plant and animal medicines used in traditional medicine in the past and present, aiming to promote the development of ethnopharmacology and is a representative journal in the field of complementary medicine. Moreover, *Frontiers in Pharmacology*, *Evidence-Based Complementary and Alternative Medicine*, and *Biomedicine & Pharmacotherapy* also were relatively productive, *Pharmacological Research* had the highest IF. These journals are internationally renowned and have greater international influence in TCM research, which can provide a reference for the publication of articles in related fields. Meanwhile, we can also see that although most TCM/CM articles were published in China, there was a lack of influential international journals, and it is necessary to improve the international influence of Chinese journals.

### 4.2 Research hotspots and trends

High-frequency keywords are often used to identify hot topics of a field, while cluster analysis is mainly used to find the distribution of main research contents under the research topics. High-frequency keywords co-occurrence and cluster analysis results showed the hotspots and main research contents in TCM/GM, mainly related to the role of GM in TCM treatment of glucolipid metabolic disease and lower gastrointestinal diseases, the mechanism of interactions between GM and TCM to treat diseases, the links between TCM/GM and metabolism, and the relation between GM and the oral bioavailability of TCM.

#### 4.2.1 The role of GM in the TCM treatment of diseases

Metabolic diseases such as obesity, T_2_DM and hyperlipidemia are associated with GM dysfunction. TCM such as berberine has shown considerable efficacy in the treatment of metabolic disorders by modulating GM (Yang et al., 2021; Zhang et al., 2021). (1) Obesity and Lipid Metabolism: Human GM associated with obesity has been studied earlier (Ley et al., 2006; Tremaroli and Bäckhed, 2012; Zhao, 2013; Shin et al., 2014). Some studies showed that TCM such as Celastrol (Hua et al., 2021), Ganoderma lucidum (Chang et al., 2015) and Daesiho-Tang (Hussain et al., 2016) can attenuate HFD-induced obesity by remodeling GM to inhibit lipid absorption. Moreover, Liu et al. (2019) showed that Coix seed can be used as a prebiotic preparation to reduce body weight and prevent obesity-related metabolic disorders. Wu et al. (2021) found that Bupleurum radix extract can improve the impaired lipid metabolism in HFD-induced obese mice through regulation of GM-mediated FGF21 signaling pathway. Er-Chen Decoction has a beneficial effect on obesity, especially lipid metabolism disorder, which is related to the regulation of GM (Zhao et al., 2021). A meta-analysis showed that oral TCM preparations could improve lipid metabolism disorders in patients by regulating GM (Gong et al., 2022). (2) T_2_DM and Insulin Resistance: Many herbal monomers and formulations can pass the “bacteria-mucosal immunity-inflammation-diabetes” axis to improve glucose metabolism and treat diabetes (Gao et al., 2017; Zheng et al., 2020). Cao et al. (2019) found that Jinqi-Jiangtang Tablet may improve the insulin sensitivity of T_2_DM mice by improving the GM and promoting the production of SCFAs. Zheng et al. (2020) showed that Bu-Zang-Tong-Luo Decoction has a beneficial effect on diabetic hindlimb ischemia by remodeling the structure of GM. Wang et al. (2019) reported that Ophiopogon japonicus tuber polysaccharide can be used as a new type of functional food to prevent diabetes-related GM dysbiosis and metabolic disorder. Berberine can ameliorate liver injury-induced glucose and lipid metabolism disorders by alleviating ER stress in hepatocytes and regulating GM (Yang et al., 2021). A meta-analysis showed that TCM could regulate GM and improve glucose metabolism in T_2_DM patients (Zheng et al., 2021). (3) NAFLD: GM is an important target for the TCM treatment of NAFLD (Ma et al., 2017). For instance, Ginsenosides can improve NAFLD through the comprehensive regulation of GM, inflammation and energy homeostasis (Liang et al., 2021). Gypenosides can modulate GM to attenuate disease progression in mice with NAFLD (Huang et al., 2019). Sheng-Jiang Powder (Li et al., 2020) and GQD (Guo et al., 2018) can alleviate HFD-induced steatohepatitis and improve NAFLD by regulating GM. A review detailed the significance of GM in the pathogenesis of NAFLD and the current status of TCM treatment of NAFLD by regulating GM (Li and Hu, 2020).

Lower gastrointestinal diseases are also a hotspot in TCM/GM research, TCM can regulate GM to treat IBD (mainly UC), irritable bowel syndrome (IBS) and Colorectal cancer (CRC) (1). **UC:** GM dysbiosis is closely related to UC, and TCM can alter the composition of GM to treat UC by enriching beneficial bacteria or SCFA-producing bacteria and reducing pathogenic bacteria (Feng et al., 2022). For example, Rhubarb Peony Decoction can restore Th17/Treg balance by regulating GM to improve UC (Luo et al., 2019). Li-Zhong Decoction can improve UC by regulating GM and its metabolites. The combined treatment of probiotics and TCM can inhibit intestinal inflammation and reduce adverse events for UC (Hu et al., 2022). (2) IBS: Li et al. (2018) showed that Tong-Xie-Yao-Fang could reduce colonic serotonin levels and alleviate the symptoms of IBS by beneficially affecting GM. Chen et al. (2017) found that Wu-Ji-Wan may relieve IBS by regulating the GM and stabilizing the intestinal mucosal barrier. (3) CRC: Lv et al. (2019) showed that GQD can enhance the efficacy of PD-1 in CRC by remodeling the GM and tumor microenvironment. A further study (Li et al., 2020) showed that GQD can modulate the GM to enhance the immunity of CRC patients and protect the intestinal barrier function. Sui et al. (2020) found that YYFZBJS ameliorated CRC progression in mice by remodeling GM and inhibiting regulatory T cell generation.

#### 4.2.2 Mechanisms of TCM to treat diseases by regulating GM

GM dysbiosis and altered metabolites can result in the promotion of endotoxin-mediated promotion of metabolic inflammation, destruction of the intestinal mucosal barrier, and decreased host anti-oxidative stress capacity, which are the main reasons for the link between GM and host diseases (Everard et al., 2013). However, excitingly, the interactions between TCM and GM may break this link by intervening in inflammation, metabolism, oxidative stress, and IMB. Many review articles (Xu et al., 2017; Feng et al., 2019; An et al., 2019; Lin et al., 2019; Zhang et al., 2019; Zhang et al., 2020; Gong et al., 2020) had outlined the interactions between TCM and GM (**Table 7**), including: (a) TCM can regulate the composition of GM and restore the balance of GM, such as increasing anti-inflammatory and SCFAs-producing bacteria and reducing pathogenic bacteria; (b) TCM can regulate the metabolism of GM by increasing the level of SCFAs, regulating bile acid (BA) metabolism, reducing the production of trimethylamine N-oxide (TMAO) and the release of inflammatory factors (Li et al., 2021); (c) GM can transform TCM compounds into metabolites and components with different bioavailability and bioactivity/toxicity and in turn improve the composition of GM; (d) GM can mediate interactions between multiple chemicals in TCM.

**Table 7:** The review articles on mechanism of interactions between TCM and GM.

No.	Title	First author	Year	Journals	IF	Partition	TC
1	Understanding the Molecular Mechanisms of the Interplay Between Herbal Medicines and Gut Microbiota	Xu, J	2017	Med. Res. Rev.	12.388	Q1	149
2	Gut microbiota, a new frontier to understand traditional Chinese medicines	Feng, WW	2019	Pharmacol. Res.	10.334	Q1	130
3	The interaction between the gut Microbiota and herbal medicines	An, XD	2019	Biomed. Pharmacother.	7.419	Q1	40
4	The interactions between gut microbiota and bioactive ingredients of traditional Chinese medicines: A review	Gong, X	2020	Pharmacol. Res.	10.334	Q1	23
5	Enhancing Clinical Efficacy through the Gut Microbiota: A New Field of Traditional Chinese Medicine	Lu, YM	2019	Engineering	12.834	Q1	16
6	Traditional Chinese Medicine and Gut Microbiome: Their Respective and Concert Effects on Healthcare	Zhang, RZ	2020	Front. Pharmacol.	5.988	Q1	12
7	Gut microbiota: a new angle for traditional herbal medicine research	Lin, LF	2019	RSC Adv.	4.036	Q2	11
8	Effects of traditional Chinese medicines on intestinal bacteria: A review	Chen, TT	2012	Indian J. Tradit. Knowl.	1.091	Q4	8
9	The Gut Microbiota and Traditional Chinese Medicine: A New Clinical Frontier on Cancer	Chen, YZ	2021	Curr. Drug Targets	2.937	Q3	1
10	Pivotal Role of the Interaction Between Herbal Medicines and Gut Microbiota on Disease Treatment	Zhao, TT	2021	Curr. Drug Targets	2.937	Q3	1

The specific mechanism of TCM regulating GM to treat diseases is complex, specifically, including the following aspects in our current research (Wu et al., 2019): (1) Anti-inflammatory: Inflammation regulation is the key to the efficacy of TCM/GM interaction. TCM can regulate the composition of GM and reduce inflammation caused by GM dysbiosis. For instance, berberine can modulate GM and inhibit the activation of TLR4 signaling pathway and the release of NLRP3 inflammasome and its cytokines (Zhao et al., 2022). GM plays an important role in anti-inflammatory function of berberine and luteolin (Cao et al., 2021; Franza et al., 2021). Kai-Xin-San can suppress the neuronal inflammation by regulating GM (Cao et al., 2020). Bofutsushosan can alter the GM by increasing *Akkermansia muciniphila*, and help reduce diet-induced inflammation (Fujisaka et al., 2020). Furthermore, the metabolism of GM also plays an important role in the anti-inflammatory effect of TCM (Piwowarski et al., 2014; Jang et al., 2019). (2) Anti-oxidative stress: There is growing evidence that some bacteria, such as *Lactobacillus*, *Bifidobacterium* and *Akkermansia*, and the bacterial metabolite butyrate have potential anti-oxidant properties, and as mentioned above, TCM can promote their increase. In addition, probiotics can reduce the level of intestinal oxidative stress through their own antioxidant enzymes and anti-oxidant metabolites, and some TCM such as mushroom polysaccharides from Ganoderma lucidum and Poria cocos (Khan et al., 2018), Fructooligosaccharides from Morinda officinalis (Chen et al., 2017) and Astragalus polysaccharides (Li et al., 2009) have probiotic-like effects and can play this function. (3) Protecting the intestinal barrier: The intestinal mucosal barrier (IMB) is the first line of defense against the invasion of commensal bacteria and pathogenic microorganisms. Under the influence of HFD, inflammatory stimulation, oxidative stress and other factors, the structure and function of IMB may be damaged, which may lead to intestinal injury (leaky-gut). Defects in IMB function are important factors leading to the development of diseases such as IBD. Some TCM can improve the IMB function by adjusting GM (Cao et al., 2019). For instance, Eugenol may enhance the IMB by increasing the thickness of the inner mucus layer through microbial stimulation, thereby preventing invading pathogens and diseases (Wlodarska et al., 2015). Scutellaria-Coptis (Zhang et al., 2020), Ginsenosides (Liang et al., 2021) and GQD (Li et al., 2020) can alleviate IMB damage by inhibiting inflammation and regulating GM.

#### 4.2.3 The connection between TCM/GM and metabolites

TCM/GM-related metabolites are key factors in the curative effect of herbal medicines (Wang et al., 2019). Metabolomic analysis is an ideal method to identify changes in TCM/GM-related metabolites (Shen et al., 2018). The interactions of TCM and GM can lead to the increase or decrease of certain metabolites and the production of new metabolites or the disappearance of certain metabolites (Feng et al., 2019). The metabolites mainly include three categories (Feng et al., 2018): (a) metabolites that are transformed from TCM by the GM, such as compound K and baicalein; (b) metabolites that are secreted by the host and modified by GM, such as secondary bile acids; (c) metabolites that are synthesized by GM *de novo*, such as SCFAs and polysaccharide A.

Herbal compounds can be transformed by the GM (type a metabolites) or metabolized into other types of metabolites in a new scaffold (type c metabolites). On the one hand, GM is involved in the metabolism and biotransformation of TCM components such as flavonoids and ginsenosides by producing specific enzymes, such as reductase and hydrolase (Dey, 2019; Zhang et al., 2020; Xie et al., 2020). TCM-related metabolites mediated by GM can have better bioavailability and bioactivity than their parent compounds. For example, human GM can efficiently metabolize baicalin with minor anti-tumor effect to baicalein with potent anti-tumor effect (Wang et al., 2015). Some metabolites of herbal ingredients influenced by GM were demonstrated to be more cytotoxic to tumor cells than non-metabolites (Kim et al., 2008) and have higher anticomplementary and antimicrobial activity (Xing et al., 2014). The GM can convert berberine to a more easily absorbed but inactive metabolite dihydroberberine through enzymatic catalysis, which is then oxidized back to berberine and incorporated into the blood to exert pharmacological activity (Feng et al., 2015). In fact, under fermentation by the GM, TCM can be metabolized into various metabolites with a wide range of bioactivities to affect its efficacy and toxicity (Piwowarski et al., 2014; Lin et al., 2021). On the other hand, GM-related metabolites influenced by TCM can have a wide range of effects on the therapeutic or side effects of TCM. SCFAs are the major *de novo* metabolites associated with TCM and the most well-studied in TCM/GM over the past decade (Feng et al., 2018). SCFAs can regulate gut hormone production, gut integrity, energy homeostasis, appetite, and immune function (Canfora et al., 2015). The therapeutic effect of TCM can be partially achieved by affecting SCFAs (Zhang et al., 2012; Zhang et al., 2021). For example, after using Scutellariae radix, Coptidis Rhizoma and their combination, SCFAs-producing bacteria were significantly enriched in T_2_DM rats, while secondary bile acid-producing bacteria were significantly decreased (Xiao et al., 2020). Xiexin Tang (Wei et al., 2018; Zhang et al., 2021) and HLJDD (Chen et al., 2018) can significantly ameliorate the GM of T_2_DM rats, increasing the abundance of SCFAs-producing and anti-inflammatory bacteria. Moreover, berberine (Wang et al., 2017), Poria cocos (Sun et al., 2019) and Indigo Naturalis (Sun et al., 2020) can increase the abundance of butyrate-producing bacteria and promote the production of butyrate (a SCFA fermented by the GM), which may explain their therapeutic efficacy.

#### 4.2.4 The relationship between GM and oral bioavailability of TCM

Notably, low oral bioavailability and bioactivity are a perplexing problem for some TCM. GM and its metabolism can directly or indirectly affect the biotransformation of TCM and reconcile the oral bioavailability conundrum of TCM (Chen et al., 2016; Xu et al., 2017; Zhang et al., 2020). This biotransformation may help explain the large interindividual variability in responses to TCM, as the composition of GM varies among individuals. Bioavailability is closely related to the blood concentration of TCM, which can directly reflect the efficacy and toxicity of most drugs and is regarded as an important factor in the evaluation of efficacy and safety.

A study (Kim et al., 2008) showed the GM may activate the pharmacological effects of TCM, and is a biocatalytic converter that converts herbal components into bioactive compounds. GM can induce comprehensive metabolism of herbal components and enhance the bioactivity of TCM such as ginsenosides (Wang et al., 2011). TCM is mainly taken orally. After the components of TCM are absorbed into the blood through the gastrointestinal tract, they are mainly metabolized in the liver, and finally generate a variety of metabolites. However, studies have shown that the bioavailability of most compounds in TCM is very low (Zheng et al., 2020). For example, the bioavailability of ginsenosides is typically around 0.1%–0.5% by oral administration (Liu et al., 2009) and the oral bioavailability of berberine is less than 1% (Chen et al., 2011). Flavonoids are key bioactive substances in herbal medicines with pharmacological potential, but their bioavailability is also low (Dey, 2019). Recent studies have shown that TCM ingredients inevitably come into contact with microorganisms in the gastrointestinal tract during oral administration, especially saponins with larger molecular weight, which are usually poorly absorbed in the intestinal tract, have low bioavailability and a relatively long retention time in the intestinal tract. Longer, more susceptible to the influence of GM. Under the action of GM, the components of TCM will undergo a series of structural changes, and the resulting transformation products may have better bioavailability or stronger biological activity than the original components (Kim et al., 2008; Feng et al., 2015; Dey, 2019). Furthermore, the potential of TCM as a therapeutic agent may not only depend on its bioavailability, but its medicinal value may also stem from its positive effects on GM-mediated gastrointestinal health (Lopresti, 2018).

### 4.3 Related research on specific areas of TCM/GM

Some articles may indicate future research trends, but there may be less chance of being cited. Therefore, we searched for articles and found representative papers among them. The specific studies are as follows:

(1) TCM Syndromes research: GM imbalance is an important part of TCM syndromes characteristic community. For example, GM imbalance and subsequent metabolic changes in patients with qi deficiency affected immunity and energy metabolism, thereby increasing the susceptibility to diseases (Ma et al., 2018). GM in patients with spleen deficiency syndrome (Peng et al., 2020), spleen-yang-deficiency syndrome (Lin et al., 2018), and kidney-yang deficiency syndrome (Chen et al., 2019) had changed, while TCM can help to restore the richness and diversity of GM in specific TCM syndromes (Zheng et al., 2020), which provided insights into the correlation between TCM syndromes and GM imbalance and a new method for further studying the pathogenesis of GM regulating specific TCM syndrome. Two studies have shown that GM can help to distinguish the two TCM syndromes of UC (Zhang et al., 2019) and colorectal cancer (Wang et al., 2020), which can be used as the biological basis for TCM syndrome differentiation and treatment of related diseases. (2) Acupuncture and Moxibustion research: Moxibustion may promote the repair of gastric mucosal injury by increasing the number and types of intestinal beneficial bacteria (He et al., 2019). Acupuncture can effectively alleviate insomnia and change the GM (Huangfu et al., 2019). Acupuncture at specific acupoints can delay the weight loss and tumor development of mice, and change the abundance of specific genera of GM (Xu et al., 2020). Moreover, acupuncture treatment may be an effective method to improve sleep disorders, and the mechanism may be related to the change of GM (Hong et al., 2020). (3) Integrative Chinese and western medicine study: GQD can enhance PD-1 blockade in CRC by remodeling GM (Lv et al., 2019). Berberine has potential benefits in combination with methimazole in modulating GM composition in the treatment of Graves’ disease (Han et al., 2021). In addition, complementary and alternative medicine prevention and treatment strategies such as TCM may help reduce antibiotic use (Baars et al., 2019). Notably, antibiotics disrupt the homeostasis of GM, thereby affecting the efficacy of TCM. Some studies have shown that TCM can reduce its efficacy when combined with antibiotics. For example, Kai-Xin-San can suppress the neuronal inflammation improved the depression-like behavior, while antibiotic treatment attenuated the antidepressant-like effect (Cao et al., 2020). Cassiae Semen had a hepatoprotective effect on NAFLD by modulating GM, and antibiotics can inhibit or eliminate this effect (Luo et al., 2021). Antibiotics can affect the metabolism and pharmacokinetics of the active ingredients of Scutellaria baicalensis, which should not be used in combination with antibiotics in clinical use (Xing et al., 2017). The method of combined application of TCM and western medicine to improve clinical efficacy still needs to be further explored.

### 4.4 Limitations of the study

This study still has some limitations. First, even though we have found the top cited articles, newly published papers may also have better influence but may be cited less frequently. Second, only the papers included in the SCI-E of WoSCC were searched, and some papers not included in the of WoSCC were not included in the analysis, which may cause some bias in the results. Third, only English literatures-included and the articles/reviews-based search strategy mean that a few articles involving TCM/GM may be ignored. Fourth, this study is only an analysis of research articles at the current stage. With the rapid development of TCM/GM research, more articles will be available for analysis.

## 5 Conclusion

In summary, with the help of scientometrics and visual analysis methods, this study preliminarily shows the global research status and trends of interactions between GM and TCM. The results show that in recent years, the research attention of TCM/GM has gradually increased. The TCM/GM research mainly focused on the effects of TCM on the GM of HFD-induced animal models, the mechanism of regulating GM imbalance to prevent and treat glucolipid metabolism diseases and lower gastrointestinal diseases, and the links among GM, metabolism, and oral bioavailability of TCM. In the future, TCM/GM research will delve into more diverse disease types, such as cancers, psychiatric and neurological disorders, and so on. Moreover, more in-depth research will work to identify specific bacteria and metabolites that affect the efficacy and toxicity of TCM, so as to better play the role of TCM treatment. All in all, GM provides a new opportunity for elucidating the mechanism of TCM in treating diseases. TCM can be also a treasure trove of potential prebiotics. GM, especially GM-related metabolites, may be key mediators linking TCM to host physiological states. Increasing explorations of TCM-GM interactions would have the opportunity to revolutionize the way we view TCM. GM-centric research could be the next breakthrough in TCM, and targeting the GM may become a new strategy for treating diseases through TCM in the future.

## Data availability statement

The raw data supporting the conclusions of this article will be made available by the authors, without undue reservation.

## Author contributions

SY: writing-original draft preparation, manuscript, investigation, and figure preparation. SH: manuscript, investigation, and figure preparation. QW: data collection and manuscript proofreading. YL: conceptualization, methodology, and supervision. LJ: investigation, methodology, and supervision. DC: conceptualization, methodology, and supervision. All authors contributed to the article and approved the submitted version.

## Funding

This work was supported by Foundation for Young Scientist of China-Japan Friendship Hospital(2019-2-QN-63), Natural Science Foundation of Beijing Municipality (7214295), National Natural Science Foundation of China (81973693) and National Natural Science Foundation of China (82104599).

## Conflict of interest

The authors declare that the research was conducted in the absence of any commercial or financial relationships that could be construed as a potential conflict of interest.

## Publisher’s note

All claims expressed in this article are solely those of the authors and do not necessarily represent those of their affiliated organizations, or those of the publisher, the editors and the reviewers. Any product that may be evaluated in this article, or claim that may be made by its manufacturer, is not guaranteed or endorsed by the publisher.
